# “HealthOmeter”: An Aid in Advancing Preventive Medicine Media Revolution

**DOI:** 10.1155/2015/798971

**Published:** 2015-11-19

**Authors:** Erik Trell

**Affiliations:** Department of Family Medicine, Faculty of Health Sciences, Linköping University, 58185 Linköping, Sweden

## Abstract

Subjective wellbeing is an important issue on the preventive medicine and political agenda and for mutual communication, information, and interaction in society and its individuals “requires new tools for measuring phenomena previously believed unmeasurable, as well as conceptual frameworks for interpreting such measurements…considering both happiness and misery.” The task is difficult, however, due to the great span of parameters and variables of age and gender, settings, socioeconomic conditions, wellness and illness, activities and functions, roles and habits, thoughts and feelings, and experiences and expectations involved over the panorama. HealthOmeter is a clinically tested and validated instrument with design and capacity in distinct coherent chapters to meet the new measurement and interpretation demands both contentwise and operationwise. Over the range of subjective and objective health it enables, in a uniform normalized layout in quintile balance between positive and negative, an all-round self-assessment and counsel in multimedia, preferably computer/mobile app distribution including storage, collation, and follow-up in full integrity and secrecy on the individual and aggregated level.

## 1. Introduction

The recent Science perspective “Progress in Measuring Subjective Well-Being. Moving Toward National Indicators and Policy Evaluations” [1] heralds a paradigm shift in the concepts and concerns of health and care in the rich fabric of human life and environment in modern affluent societies. Also the important policy reverberations and challenges of that shift “refer to how people experience and evaluate their lives and specific domains and activities in their lives” where “progress in science requires new tools for measuring phenomena previously believed unmeasurable, as well as conceptual frameworks for interpreting such measurements” [Ib.] both on an individual and on aggregated basis.

There are fundamental factors and forces rooted in the far-reaching political changes and information revolution of our time which more and more move the top-down central management “in policy evaluation and decisions” to the ground-up thoughts and feelings and perceptions in the population and how to tune in and stay in touch and contact with the genuine will and interests of the people, “because individuals and policymakers value subjective outcomes and because such outcomes appear to be affected by major policy interventions” [Ib.]. As expressed in the Science perspective, this poses new challenges in relation to health, life quality, and “hedonic wellbeing” [Ib.] not only for governments and planners but equally much for the medical services. There is a well-known “Health Paradox” in affluent societies; that is, the higher and more prioritized and centrally delivered the technological standards in superspecialized care are, the higher the population's dissatisfaction with the general health situation tends to be [2]. Unless preventive medicine's compass and programs “include the mental/subjective and the functional/elderly care sectors as well as life quality, wellbeing, and related spheres” in direct involvement with the citizens this paradox will not resolve [3].

In a similar vein, the Science position paper concludes that “emerging evidence finds that self-reports are related to biological processes and health…especially important is progress on both fronts in the measurement of subjective wellbeing (SWB)…Earlier this year the National Research Council of the U.S. Academy of Sciences issued a report on hedonic wellbeing and policy. This stressed the importance of considering both happiness and misery…we should measure wellbeing more often and do it comprehensively…this would help governments improve policies, companies raise productivity, and people live more satisfying lives…measures of SWB are likely to play an increasingly important role in policy evaluation and decisions” [1]. For many years our group has been working along these lines from a preventive medicine angle [3, 4], widening out to broader population cooperation [5]. Our experiences might be of interest and applicability in the described larger context.

## 2. Methods

### 2.1. HealthOmeter Construction

**Figure 1:**
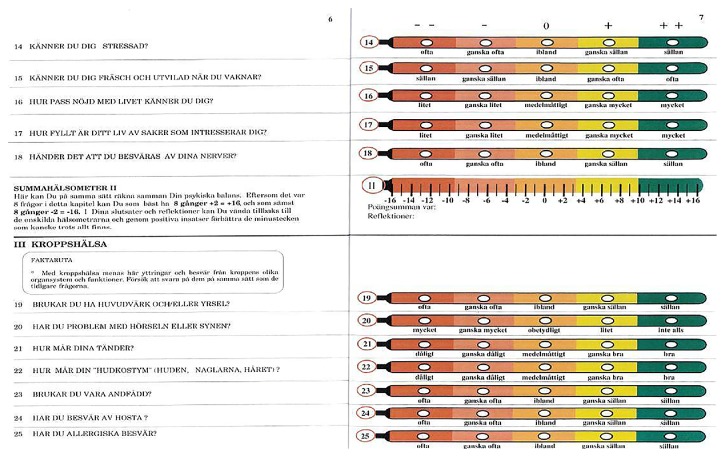
In Swedish. A page from short screening questionnaire used in the Östergötland county population health policy program [5] with concluding part of mental health chapter and its summing-up scale and start of physical health chapter with its short introductory fact box. The largely self-explanatory summing-up method is to detract the amount of minus signs from and add the amount of plus signs to the grand total. In interactive computer and app versions this can be continuously supported and reinforced including interpretation, conclusions, and advice.

The Malmö Preventive Program was pioneering in interactive multiphasic health screening and intervention on a whole-community basis [4] and probably first in the world to develop and implement an online distributed computer system for managing both the comprehensive biometric part of the examination by the staff [6] and the penetrating questionnaire of 260 items by the attenders themselves [7]. To increase their focusing on the self-report and enhance its pedagogical effects, it was completed in privacy in reclining rest via separate terminals in each of twelve “cubicles” during the 2-hour oral glucose tolerance test performed there [4, 7]. In assessments as well as reports of findings and results, a common, epidemiologically optimal quintile-distributed scale was applied for both subjective and objective and gradual and discrete values. Thus the format was found for self-dispensation of the program, as later realized in other settings by the HealthOmeter (Hälsometer^©^, Helsemeter, Hygeiometro, and Gesundheits-Thermometer) here described. Its construction is recapitulated from a previous dissertation study report [3] as follows: “Based upon the experience of the Department of Preventive Medicine in Malmö, Sweden, a self-mediated, interactive health testing and promotion instrument called “HealthOmeter” has been developed and tested for feasibility. The instrument uses a special variety of a quintile-distributed visual analog scale (VAS) (Figure 1) with the thermometer as reference and allows (a) easy summation and averaging of single or different aggregates of the test items as a “wellness profile” and score with emphasis upon the positive aspects of health and (b) recognition against this on the whole favorable background of the weak points motivating further action. The instrument, which can be distributed on paper or electronic medium, supports participation and insight in the initial stages of a directed individual health program, for the continuation of which the utilization of professional counsel is stimulated” [Ib.].

### 2.2. Face and Contents Validation

Regarding the validity, reliability, and performance tests of the instrument [8], it is important to point out that it comprises already established items and variables well investigated and documented in the literature and in many instances by studies on site [3–8]. When in the present case specifically concerning validity and reliability of operative feasibility and comprehension, the Delphi method was applied in a sizable nominal group [8]. The Nominal Group Technique is particularly useful in design and monitoring of education curricula and programs [Ib.] and gave positive opinions and suggestions related to three main areas of instrument qualification, that is, hardware, software, and “wetware,” which are here presented together with our own conclusions and experiences and examples of carried-out appliances.

## 3. Results

### 3.1. Hardware

**Figure 2:**
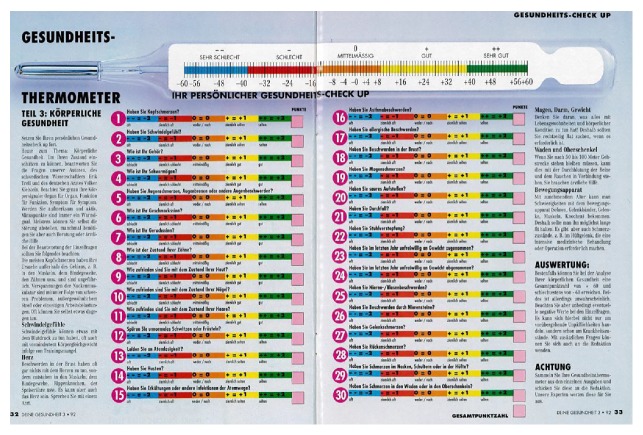
Physical health Gesundheit-Thermometer in issue of Deine Gesundheit, with summing-up scale on top.

The hardware is more than just mechanical parts but in effect crucial to provide a good platform for the interaction. Especially in a communication context there should be inspiration and feedback for getting involved, informed, and empowered. The media may not be the message but must convey the message properly and durably. To that end, paper is still, and remains, well suited and, above all, stable. It can be saved and repeated as a logbook. It can be given a wide variety of designs, from questionnaires and brochures to newspaper columns. An example of the latter is the* Gesundheits-Thermometer*, “Ihr persönlicher Gesundheits-Check Up” (Figure 2) of in all 138 items run as a series over the different health chapters in consecutive issues of the popular German “Deine Gesundheit” magazine.

Another example is the Hälsometer booklet of 135 items specially composed for and used in a national campaign of elderly health in Sweden [3] where extra emphasis was put upon social conditions, activities, and functions of daily life, and dietary and nutritional factors, all of which become more and more vital when years go by. It brings to mind that there is a double three-dimensionality of health which has to be taken into consideration in the practical appliance. First there are the three WHO dimensions of physical, mental, and social health which at least in approximation can be considered as linearly independent and thus spanned over a Cartesian coordinate space embracing both positive and negative directions of the green *x*-, blue *y*-, and red *z*-axes assigned for them in that order. And then there are the more gradual dimensions of young-age health, middle-age health, and old-age health, which are dependent, in the sense of being predominantly related to the *y*-, *x*-, and *z*-axes, respectively. In other words, it is not fruitful to ask a teenager about the use of hearing aids and walking sticks, but more about music, sports, good feelings, peace, and friendship, which are of course vital in the other ages, too, but in another blend. All may be transferred to and by the HealthOmeter representation, however, supporting its flexible directed usefulness under the new signals and challenges [1].

And while the paper forum is still highly applicable, for instance, in a nutrient-, vitamin-, and mineral-oriented health cookbook with a rhapsody of interpolating illustrations and recipes, new media have appeared that are in some respects even better suited for HealthOmeter conveyance. The interactive computer was used from the start of the Malmö Preventive Program (and later also in two HealtHotel^©^ installations) [3] and has the given advantage but also integrity problem of its practically infinite processing, storage, and network capacity. It is still unsurpassed both in personal and public services and naturally aimed at an optimally user-friendly on-line dialogue. Yet, it is the smart mobile phone and its apps and games that are finally getting close to offer the perfect communication means, especially for the coming generations. And so it happens that when HealthOmeter has now come of age, the ultimate hardware revolution brings it back to the future because of its suitability for app and similar implementation.

### 3.2. Software

**Figure 3:**
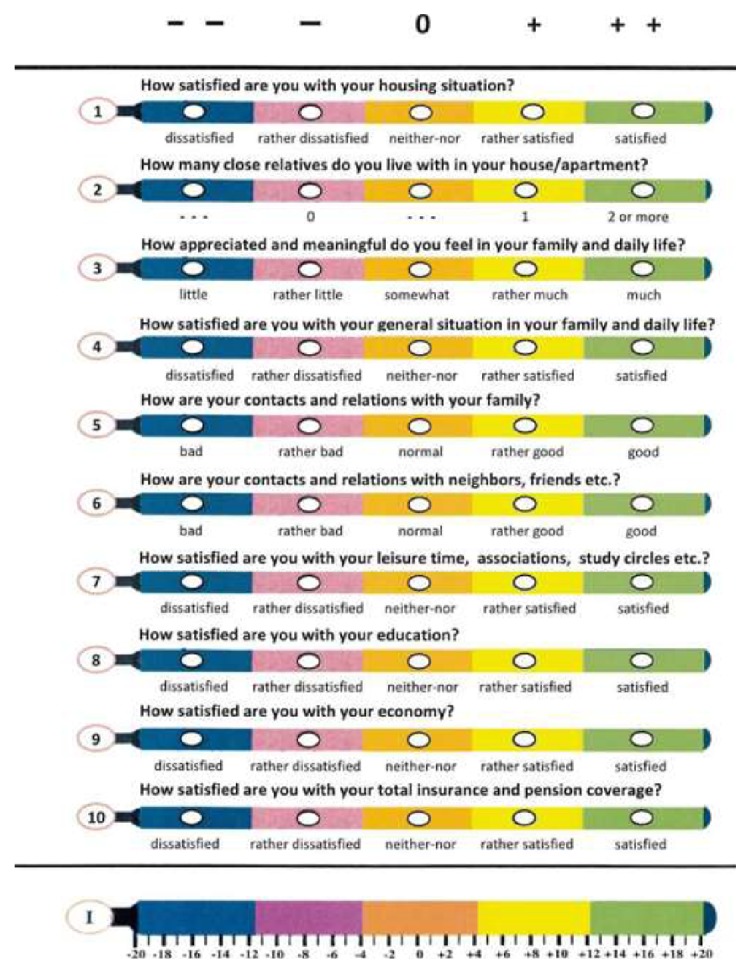
English translation of ordinal VAS scales in the social health section of the elderly health version.

**Figure 4:**
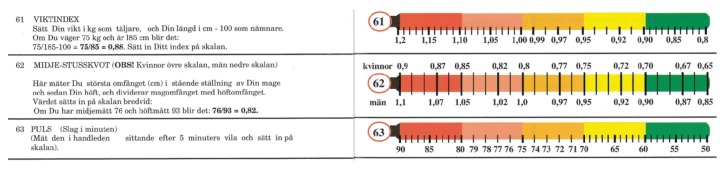
Numerical scales of self-assessed weight index, waist-to-hip ratio, and pulse in their actual population sample quintile distributions. In, for example, on-line internet/cloud implementations these can be automatically calibrated and age- and sex-standardized for the user* pari passu* with the global accumulation of data.

The software, that is, program structure, is based upon the normal distribution curve of variables, fit into quintile order. This is the most common epidemiological standard when it comes to biometrical measurements and their interrelations and outcome correlations. Higher resolutions, for example, deciles, may often blur and weaken the predictive as well as identification power and have less evidence basis in the literature and in addition lack a zero or normal balance pivotal midpoint. And lower resolutions, for example, tertiles, are too insensitive. Furthermore, the same arguments largely apply to ranking, or ordinal scales, too, which are those used in “vignette” [1] and VAS and other types of subjective as well as objective qualitative self- (Figures 1–3) or observer registration (Figure 4) and in this way may be tallied with the numerical values and so together equip the unified HealthOmeter presentation. The almost obvious extra twist of this is to operationalize the fact of the equal positive and negative tails of the distribution, tending to be overlooked in many “Health Curve” appliances which are usually inclined towards the negative, risk portion of the spectrum. But health is bridged over good through bad and in fact by the biological powers of life more to the positive side since most of us after all feel well. In these regards HealthOmeter responds to the novel requirements of “progress in measuring…as well as conceptual frameworks for interpreting such measurements…considering both happiness and misery” [1].

The user comprehensiveness and participation are spurred by the “vignette approach” [1], here in the form of a VAS with the thermometer as reference as “explicit comparison standard” (Figures 1–4) [Ib.]. Accompanying audiovisual information and interpretation inserts (Figures 1 and 2) further increase the engagement, and the identification of one's “health temperature” is facilitated by harmonizing color in each VAS (Figures 1–4). A “comprehensive national indicator” (Ib.) must be able to combine “several fundamental nonoverlapping aspects such as material…and emotional…verbal…and numeric rating scales [into] a coherent framework” [Ib.] (Figures 1–4) over the compass of health and wellbeing. This is achieved in HealthOmeter by the division in distinct but interrelated chapters and, as mentioned, by the use of quintile distribution over the adjusted normal range as strongly scientifically backed because it is the standard epidemiological way of representing and analyzing biometrical (Figure 4) as well as rating data (Figures 1–3) [3, 8–13]. Furthermore, the constant use of quintiles besides providing a zero pivotal point for balancing and summing up the individual and variously aggregated outcomes naturally installs a uniform matrix structure enabling instant computer storage, processing, and short- and long-term compilation and audit.

### 3.3. “Wetware”

This term has found use in informatics as referring to the quality and relevance of a program. In other words, no matter how fine its hardware and software are, it is of no good when processing but pointless questions. “How strong are you?” and “How beautiful are you?” are senseless as many other self-assessments one may see on the bustling vanity fair. In serious preventive medicine and national and executive standards only the best will do. The strength of HealthOmeter, that is, its “wetware” and what it holds and does, has been derived and tested from acknowledged sources at the Department of Preventive Medicine in Malmö and in some instances even originated there. Examples are alcohol [9–11] and smoking related cancer prevention [12], but also the dietary habits, glucose intolerance, overweight, social and psychological health, and other factors were optimized and integrated in eight “health chapters” [Table 1] so that even according to the most qualified big science requirements today HealthOmeter's “innovative new work provides a framework in which individuals' utility depends on several nonoverlapping aspects…and components could be aggregated…(into)…a coherent framework for aggregating dimensions of SWB” [1].

Although in-depth discussion is out of the scope here, a few comments are warranted, starting with the social factors. They form a seemingly rigid framework, but that does not mean that they are fixed. On the contrary, many of them involve subjective feelings and reactions and are therefore amenable to own or assisted improvement. This is true in all ages, not the least in the old, as reflected in the social health chapter (Figure 3) of the* Hälsometer* for the elderly. Most of the variables there can be influenced and demonstrate that subjective wellbeing and coping and their “self-reports are related to biological processes and health” [1] in a communicative and tractable sense. The ratings with their synthesis of information and feedback assume a second dimension with both medical and policy purport.

Beyond the entry stage each chapter can be expanded into deepened consultation support by the same approach. As particularly evident in the mental health field it is important to keep the boundary between the SWB screening and the clinical management levels. In the first frontline it is about getting aware of the subject and opening up personally relevant aspects (and freeing from others). This applies in the ensuing physical health inventory, too (Figure 2), where recognized patterns of subjective symptoms likewise are guide to matching professional attention.

The quintessential, diet, physical activity, leisure-time, tobacco, and alcohol, life habits sphere is different insofar as its great variety is largely left to personal control and coaching. It is therefore even more calling for elucidation. For instance, the dietary and nutritional factors are vital in the elderly [13] and in the designated Hälsometer are covered by special questions on cooking and eating habits and ten questions each on salt, sugar, fat, and fibers/vitamins/minerals. And similarly multiplying the power of the alcohol and tobacco questions, they can be increased in number, adding to the positive health impact of never having drunk/smoked or to the negative weight of heavy consumption.

Medication, health care attitudes and utilization, and hereditary factors also contribute to the integrated health profile and awareness. The normal result in every chapter is to come out with a net plus score and in that after all positive health situation one is able to address the weak points with more confidence. For instance, heredity for diabetes is not doom but operated by factors, for example, of diet or physical inactivity or overweight possible to correct; it is better if they are recognized early. The steep slope of the normal distribution means that there is a short step between adjacent positions, on the scale as well as in reality.

The biometrical variables, finally (Figure 4), are a complement rather than autonomous determinants. The exception is blood pressure which also when measured at another occasion or already known can be brought to the (in computer and app versions automatically) age- and sex-adjusted scale. The other physical data, weight index, waist-to-hip ratio, and pulse, can be measured by oneself and have corresponding subjective rating sites in the HealthOmeter (Figure 4). Only two chemical analyses are included in the ground version, blood lipids and glucose. It is possible to identify single or clusters of answers in the various chapters giving stronger or weaker recommendations to perform either or both of them.

Again it is affirmed that “self-reports are related to biological processes” [1] and that HealthOmeter serves well in self-administration. In individual cases this is sufficient but large-scale projects need resourceful management centers of excellence for running and upgrading the program. This is true also when the HealthOmeter principles may be expanded into clinical sectors and programs and even beyond the medical sphere, for example, examining the corporate climate and state of health of a company or administration.

## 4. Discussion

The glittering fitness industry consciously exploits the media to incite and profit from the consumers' positive self-identification in its carnival mirror. The nonaccountable commerce is winning market shares and popularity while the regular health services lose momentum in spite of superior professional skills, institutional resources, and supplies. This is not a trivial matter but a serious issue of getting distanced from the public dialogue and interaction, with repercussions beyond the medical systems even up to the governmental level [1]. And the paradox is that it happens in an era when people thanks to the media revolution are better informed than ever.

In this situation there should be, and there is [14], a large interest and demand in society for the best provider, from which, however, there is an understandable reluctance to enter the race. There are, for example, problems of responsibility and integrity that are different from those of the more casual competitors. But the most essential reservation relates to the fundamental bioethical principle of* primum non nocere*, which has kept both preventive and curative medicine occupied with the negative, that is, risk and disease part of the field, and to the noble mission of working there in order first of all to avoid and relieve harm.

However, as quoted in Epidemic [15], Hippocrates' own precept was “to do good or to do no harm.” Good and do good relate to the positive side of a range, and the general epidemiological evidence is of equally strong correlation of good values of an indicator with favorable outcome in that domain as of bad values with unfavorable. Translated into the plus and minus points in quintile ordering of HealthOmeter's patchwork, this shapes up a fit wellbeing panacea (*panakeia*) to bring it out among (*epi*) the people (*demos*) according to the original Hippocratic canon.

What constitutes subjective wellbeing is feeling good and being satisfied, as it is possible to objectively specify it as plus scores in the scientifically validated rating scales in the respective patches. All of these, except course smoking and alcohol when present, are bound to come out with net positive in the ordinary person which from this still intact position can recognize the weak points and their pattern and start doing something about them, as usually hinted at in the passing. And the good sides are as well improved thereby and can always be strengthened separately at a later stage, too. A latent but forgotten benefit is opened, namely, raising life quality and wellbeing, as a delivery and contract. It is an achievement aspect that is often overlooked in health care: the direct and autonomous offer of bringing life to years. And years to life are also looming in the transaction. It is our experience that when people start working with this “quintile quiz” (of the “colorful quilt of health”), they gradually get more and more engaged parallel with their noticing of the pedagogical build-up of knowledge and that it pertains just to them and on the whole adds to a handsome portrait where the occasional beauty spots can be attended in full rapport. The somewhat colloquial tone and terms above echo that in its sequential build and presentation the session becomes a kind of personal problem-oriented [16] “game-of-health” learning adventure conditioned also by the thermometer vignette and the harmonizing tinge of this association-rich visual analog scale. Especially in interactive computer/app operation all of this of course possibly varies indefinitely in sequence and layout of presentation, support, and feedback.

A legitimate reservation may now exist if such a system is Columbus' egg or Pandora's box, or both. However, it only spans over the normal numerical and expression range of the included variables, and excesses on both sides are guided to seek further counsel. Moreover, the variables are nontrivial since singly or in combination they are carrying a risk in their negative end and reciprocally, and not only by inference but documented scientific evidence, a positive influence in the other. While thus “considering both happiness and misery” (1), the balance is set to come out well on the plus side in the grand total. Practically everybody using it will be able to recognize their own complexion there as belonging to the ordinary wholesome crowd. Again, people like and deserve to feel positive and together, not the least when in some respect seeking assistance. A natural communication forum is established facilitating contact and exchange on the local or optionally larger plane. The homogenous matrix structure means overall compatibility and computability in the quintile format, that is, the veritable* lingua franca* of epidemiology and preventive medicine alike. When by app or computer HealthOmeter is performed directly in the internet, an information bank in neuronal network form will accumulate that may reach large size and enable valuable statistical and other comparisons. Full individual confidentiality and strictly personal access and retrieval can still be safeguarded at any level, while unidentified subsets can also be locally processed to both the own and common good.

The bearings to the Science perspective [1] on “how people experience and evaluate their lives and specific domains and activities in their lives…in which individuals utility depends on several, nonoverlapping aspects” could be the actual substance of “a credible, comprehensive index” and how such “a coherent framework would help governments improve policies, companies raise productivity, and people live more satisfying lives” is manifest. Instead of an alienated top-down counting of more and more distant debit figures in retrospect it can be an on-line sharing in informed consent and confidentiality of the ground up advancement of preventive medicine and the general health services into the expanding universe of equally objective and subjective wellbeing. The anonymous data can be instantly collated with the statistics in other engaged offices, private or corporate, communal or state, demographic, social, environmental, and so forth.

That is not to split the agenda but to apply and enrich the “new insight into public health and preventive medicine” which the recently formulated “multilevel and…multidisciplinary analysis approaches” to avail for all parties offer to resolve what “may drive the health disparities” [17]. HealthOmeter brings the wellbeing into the equation, complementing it also with the positive ends that drive in the good direction, which is ever so important to deal with for the health workers and the planners and administrators, plus, last but not least, the researchers, for “progress in science” [1], too, where the situation is extra precarious today with a wide “opinion gap between scientists and the general public,” so that it is in fact a question of destiny that the “gap must not be allowed to swell into an unbridgeable chasm,” to which end “the only recourse is to have genuine, respectful dialogue with people” under guaranteed integrity and secrecy [18].

This again shows that it is a vital matter to enter and by superior quality and virtue reclaim the market, whose commercial mechanisms and merchandise when not challenged will gobble up also health and wellbeing into those alienated data galaxies where people instead of caring for individuals become targets for directed sales campaigns. “The end of privacy” [19] is here and “ways to gain patients' trust and collaboration” [Ib.] anew must be found. It is very possible that some regulation is inevitable [Ib.] but the best way is the personalized concern by the regular health services adequately updated for the task. In particular this applies to the most needing groups in society that do not form attractive trade segments, such as the old. The interest and the readiness in the population are there and on the rise as we could confirm when going out with a single-item question on something supposedly so sensitive and taboo as cognitive impairment in all inhabitants ≥ 75 years of age in a whole health care district and gained a staggering 87% participation rate [14]. And that is not to be seen as exceptional but representative of the great mission waiting to be fulfilled at large.

## Figures and Tables

**Table 1:** Health chapters and number of items in them in short screening and elderly health versions of HealthOmeter.

Health Chapter	Number of items	
	Short screening	Elderly health
(1) Social health	10	10
(2) Mental health	8	10
(3) Physical health	16	30
(4) Life habits	15	60
(5) Medicines	5	6
(6) Health and care attitudes and utilization	3	6
(7) Family health	3	4
(8) Biometrical values	5	9
Sum	65	135
